# Autoimmunity to Tropomyosin-Specific Peptides Induced by *Mycobacterium leprae* in Leprosy Patients: Identification of Mimicking Proteins

**DOI:** 10.3389/fimmu.2018.00642

**Published:** 2018-04-03

**Authors:** Itu Singh, Asha Ram Yadav, Keshar Kunja Mohanty, Kiran Katoch, Prashant Sharma, Vinay Kumar Pathak, Deepa Bisht, Umesh D. Gupta, Utpal Sengupta

**Affiliations:** ^1^Department of Immunology, National JALMA Institute for Leprosy and Other Mycobacterial Diseases, Agra, India; ^2^Department of Biochemistry, National JALMA Institute for Leprosy and Other Mycobacterial Diseases, Agra, India; ^3^Clinical Division, National JALMA Institute for Leprosy and Other Mycobacterial Diseases, Agra, India; ^4^Stanley Browne Laboratory, The Leprosy Mission Trust India, TLM Community Hospital, Delhi, India; ^5^Animal Experimentation Laboratory, National JALMA Institute for Leprosy and Other Mycobacterial Diseases, Agra, India

**Keywords:** leprosy, myosin, epitopes, mimicking proteins, autoimmunity, tropomyosin

## Abstract

**Background:**

It has been shown earlier that there is a rise in the levels of autoantibodies and T cell response to cytoskeletal proteins in leprosy. Our group recently demonstrated a rise in both T and B cell responses to keratin and myelin basic protein in all types of leprosy patients and their associations in type 1 reaction (T1R) group of leprosy.

**Objectives:**

In this study, we investigated the association of levels of autoantibodies and lymphoproliferation against myosin in leprosy patients across the spectrum and tried to find out the mimicking proteins or epitopes between host protein and protein/s of *Mycobacterium leprae*.

**Methodology:**

One hundred and sixty-nine leprosy patients and 55 healthy controls (HC) were enrolled in the present study. Levels of anti-myosin antibodies and T-cell responses against myosin were measured by ELISA and lymphoproliferation assay, respectively. Using 2-D gel electrophoresis, western blot and MALDI-TOF/TOF antibody-reactive spots were identified. Three-dimensional structure of mimicking proteins was modeled by online server. B cell epitopes of the proteins were predicted by BCPREDS server 1.0 followed by identification of mimicking epitopes. Mice of inbred BALB/c strain were hyperimmunized with *M. leprae* soluble antigen (MLSA) and splenocytes and lymph node cells of these animals were adoptively transferred to naïve mice.

**Results:**

Highest level of anti-myosin antibodies was noted in sera of T1R leprosy patients. We observed significantly higher levels of lymphoproliferative response (*p* < 0.05) with myosin in all types of leprosy patients compared to HC. Further, hyperimmunization of inbred BALB/c strain of female mice and rabbit with MLSA revealed that both hyperimmunized rabbit and mice evoked heightened levels of antibodies against myosin and this autoimmune response could be adoptively transferred from hyperimmunized to naïve mice. Tropomyosin was found to be mimicking with ATP-dependent Clp protease ATP-binding subunit of *M. leprae*. We found four mimicking epitopes between these sequences.

**Conclusion:**

These data suggest that these mimicking proteins tropomyosin and ATP-dependent Clp protease ATP-binding subunit of *M. leprae* or more precisely mimicking epitopes (four B cell epitopes) might be responsible for extensive tissue damage during type1 reaction in leprosy.

## Introduction

Infectious agents of the environment are known to play a role in induction of an imbalance in the homeostatic mechanism of the host leading to an autoimmune disease (1). Hansen’s disease (leprosy) is a chronic granulomatous disease caused by *Mycobacterium leprae* (*M. leprae*). *M. leprae* is an obligatory intracellular bacterium. The three cardinal signs used for diagnosis of leprosy are the presence of anesthetic skin lesion(s), enlarged peripheral nerve(s) and presence of acid-fast bacilli in the skin smear (2).

Upon entry into the host, *M. leprae* is selectively phagocytosed by non-professional phagocytic cells (MHC class II negative Schwann cells) in the peripheral nerve and grow taking advantage of immunologically privileged site (3, 4). In an endemic population, about 95 (5) to 99% (6) of infected individuals do not develop any overt disease. However, it has been found out to be very infectious in household contacts of lepromatous leprosy (LL) due to repeated exposure to *M. leprae* infection (7). The host immune response is responsible for disease manifestation and progression of leprosy.

Infection may initiate a continuous antigenic stimulus and may breakdown the tolerance of the host through several non-specific mechanisms leading to autoimmunity (8). Infection with *M. leprae* evokes considerable changes in the humoral immune system, which involves aberrant responses, often associated with autoimmune syndrome. Presence of some antigenic structures of *M. leprae* that can be immunogenic and are cross-reactive to self-proteins might be responsible for the growth of *M. leprae* in lepromatous type of leprosy (9) wherein T cell-mediated immunity to *M. leprae* is virtually absent. On the contrary, in tuberculoid leprosy and during type 1 reaction (T1R), these similarities may lead to a heightened T-cell response and extensive granuloma formation while *M. leprae* is not observed in the host tissues (9). Our group also reported the sharing of mimicking B cell epitopes between *M. leprae* and the cytokeratin-10 (10) and myelin basic protein (11) of host.

In leprosy patients, impairments of nerve and muscle functions are very common. More than 20% of leprosy patients have been shown to have motor deficits and paralysis of muscles (12). Further, *M. leprae* has been shown to be present between the striated muscle fibers of both tuberculoid and lepromatous patients (13–15). *M. leprae* was also shown to be present in smooth muscle fibers of skin, lips, and nipples in LL (16). Degenerative changes in muscle identified as “Leprous myositis” have also been reported (17, 18). Based on the above literature, we hypothesized that muscle weakness in leprosy patients might be due to presence of anti-myosin antibodies, and therefore, auto-reaction might play a role in muscle damage leading to loss of muscle functions in leprosy patients. Hence, we searched for the presence of mimicking protein/s between host myosin and *M. leprae*.

## Materials and Methods

### Antigens

Non-irradiated *M. leprae* bacilli derived from armadillo was obtained from Colorado State University, Fort Collins, CO, USA (WHO Contract Number NIH-No1-AI-25469, Leprosy Research Support). *M. leprae* soluble antigen (MLSA) was obtained by sonication of cells of *M. leprae* according to published protocol (19). The protein content of MLSA was assessed by Bradford method (20). Myosin protein from porcine muscle (Cat. No. M0273) was acquired from Sigma-Aldrich Pvt. Ltd., USA.

### Study Subjects

#### Human Subjects

A total of 169 leprosy patients were enrolled from the Outpatient department of National JALMA Institute for leprosy and Other Mycobacterial Diseases (NJIL&OMD) (ICMR), Agra for the study. Patients were categorized based on Ridley and Jopling scale (21) and were grouped as borderline tuberculoid (BT) (*n* = 30), borderline borderline (BB) (*n* = 23), borderline lepromatous (BL) (*n* = 39), LL (*n* = 32), BT patients with T1R (*n* = 25) and BL/LL with type 2 reaction or erythema nodosum leprosum (ENL) (*n* = 20). Healthy students and staff of the institution with no evidence for leprosy and any other disease were taken as healthy controls (HC) (*n* = 55) in the study.

This study was approved by Institute Human Ethics Committee, and all the subjects were enrolled after giving a written consent to participate in the study.

#### Animals

Outbred female New Zealand white rabbits and female mice of inbred BALB/c strain were obtained from the Central Drug Research Institute (CSIR), Lucknow. All the animals were kept in specific pathogen-free conditions in the Department of Animal Experimentations, NJIL&OMD, Agra, India. Present study was approved by Institute Animal Ethical Committee, and we followed the guidelines laid down by Animal Research Ethics Board at our institute.

### Animal Experimentations

#### Hyperimmunization of Rabbit

Rabbits (*n* = 3 in each group) were hyperimmunized with protein concentration of 250 μg of MLSA emulsified with Freund’s incomplete adjuvant (IFA) and 250 μg of porcine myosin to produce polyclonal antibodies against these proteins. Control group of rabbits (*n* = 3) was administered with normal saline emulsified with IFA. All the animals were boosted weekly with the same dose of antigens up to eighth week.

#### Hyperimmunization of Mice

Mice (*n* = 15) were hyperimmunized with 25 μg of MLSA and control group mice (*n* = 10) were inoculated with normal saline as described earlier (10).

#### Adoptive Transfer

Cells from hyperimmunized mice were adoptively transferred to naïve female mice as reported earlier by Singh et al. (10). Briefly, adoptive transfer was done in control group (*n* = 5) by intravenous (i.v.) inoculation into the tail vein of suspensions of splenocytes and lymph nodes cells obtained from control mice. Similarly, experimental group (*n* = 5) were inoculated with immune cells acquired from MLSA-hyperimmunized group. Third group (*n* = 5) was inoculated intravenously with T cells separated by nylon wool (22) taken from MLSA-hyperimmunized group.

### Assessment of Anti-Myosin Antibodies by ELISA

#### Human Sera

ELISA was done for porcine myosin (Cat. No. M0273, Sigma-Aldrich Pvt. Ltd., USA) -reactive antibodies according to previously described protocol (11) with some changes. Porcine myosin (5 μg/ml) was coated into 96-well ELISA plate (flat bottom, Nunc Maxisorp, Denmark). ELISA was done according to previously published protocol (11) The absorbance was taken at 492 nM using Spectramax-M2 Reader (Molecular Devices, USA). The cutoff OD was calculated by adding average OD obtained in HC summed up with the value of twice SD.

### Experimental Animals

ELISA protocol used for sera from experimental animals was same as described above under human sera except some minor changes in reagents.

#### Rabbit

Peroxidase conjugated anti-rabbit IgG (Sigma-Aldrich, USA) was used as secondary antibody.

#### Mice

Dilution of plasma was 50-fold, and secondary antibody was anti-mouse IgG peroxidase (Sigma-Aldrich, USA).

### Effect of Myosin on Lymphoproliferation Assay

Lymphoproliferation assay was done as per the protocol described previously with some changes (10). Briefly, peripheral blood mononuclear cells were cultured in RPMI 1640 with 5% FBS in triplicate in presence of 10 μg/ml porcine myosin in Nunc-tissue culture plates (Denmark) and incubated in CO_2_ incubator for 5 days (Forma Scientific Inc., USA) at 37°C with 5% CO_2_ in air. Positive control culture was done with phytohemagglutinin. Cells were pulsed with 1 μCi/well of [^3^H] thymidine after 5 days and incubated further for 18 h. Skatron cell harvester was used for harvesting the cells. Liquid scintillation counting (LKB Wallac, Finland) was used to determine the radioactivity incorporated into DNA. Stimulation index (S.I.) was calculated by using following formula:

$$\begin{array}{l} \text{S.I.}=\text{Counts per minute }(\text{CPM})\text{ of stimulated cells}/\text{CPM of unstimulated cells},\\ \text{S.I.}>2\text{ was taken as significant stimulation}. \end{array}$$

### Identification of Cross-Reactive Proteins Between Porcine Myosin and MLSA

#### Characterization of Cross-Reactive Proteins

Two-dimensional PAGE, isoelectric focusing, was carried out using the protocol described by Gorg et al., 2000 (23). Protein samples (100 μg of porcine myosin/MLSA) were loaded on IPG strips (Bio-Rad Laboratories, USA) of pH 3–10 for myosin, pH 4–7 for MLSA and length 7 cm. Proteins were separated in second dimension using 10% SDS-PAGE and transferred to nitrocellulose membrane (NCM) (24). Blotted NCM was blocked with 3% BSA (Sigma, USA) for 1 h, then incubated with pooled leprosy patients’ sera (1:50) while NCM of separated proteins of MLSA was incubated with Myosin-hyperimmunized rabbit sera (1:50). These NCMs were incubated overnight at 4°C followed by three times washing with PBS containing 0.05% Tween-20 and incubated with peroxidase conjugated anti-rabbit IgG (1: 10,000) (Sigma-Aldrich, USA) for 1 h. Later, visualization of antigen antibody reactivity was done by color development with diaminobenzedine (Sigma, USA) solution. Capturing of image was done by Chemidoc (Bio-Rad Laboratories, USA).

#### MALDI-TOF Analysis

In-gel digestion with trypsin (25) was done according to previously published protocol (10). Mass spectra of digested peptides were analyzed using Mascot Wizard program (Matrix Science, Ltd., London, United Kingdom^1^). Peptide mass fingerprint of cross-reactive protein of porcine myosin with pooled leprosy patients’ sera was submitted to Mascot search engine and search parameters used for the identification were peptide mass tolerance ±30 ppm, peptide charge state 1+, and maximum missed cleavages 1. However, search parameters used for the identification of the cross-reactive protein of MLSA by MS/MS ion search was peptide mass tolerance ±100 ppm, fragment mass tolerance ±0.5 Da, maximum missed cleavages 1.

#### B Cell Epitope Prediction

BCPREDS server 1.0 was used (aap prediction method) to identify B cell epitopes of the mimicking proteins.^2^ Predicted B cell epitope length was of 20 amino acids and classifier specificity used was 75% (26).

#### Three-Dimensional Structure of Identified Protein

Structure of mimicking proteins of *M. leprae* and porcine myosin was predicted by submitting the sequence to Phyre2 server^3^ (27). VMD viewer^4^ was used for analysis of modeled structure (28).

### Statistical Analysis

Data were analyzed using GraphPad prism software version 5.0 (GraphPad Prism, La Jolla, CA, USA). Cutoff value for ELISA data were expressed as mean ± 2SD and *p* value < 0.05 was considered as statistically significant. Under the respective figure or table legend specific test used for analysis has been mentioned. PD Quest Software (Bio-Rad, USA) was used to analyze 2-D blot data.

## Results

### Levels of IgG Antibodies Against Myosin in Leprosy Patients’ Sera

**Figure 1:**
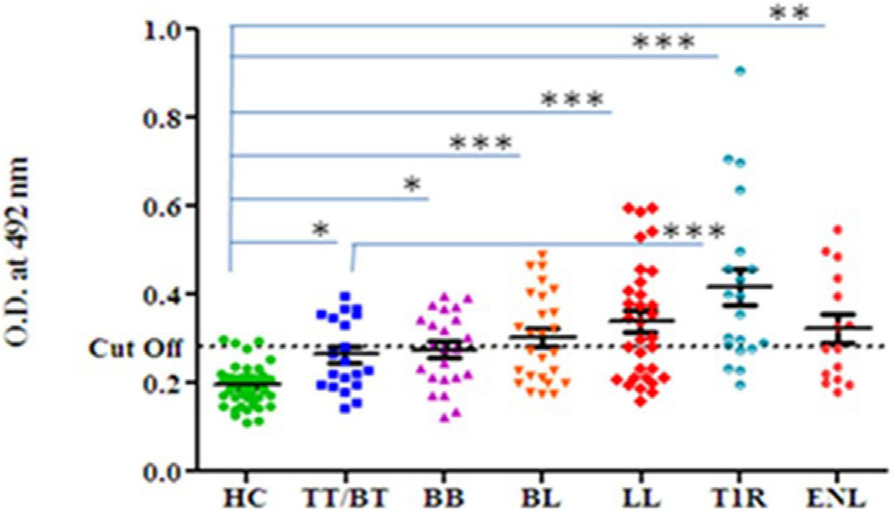
Level of antibodies against myosin in leprosy patients across the spectrum and healthy controls (HC). Dotted horizontal line represents the cutoff OD value. Smooth line with vertical lines represents the mean OD value with SEM of each group. Each dot represents the OD value at 492 nm obtained from each individual. One-way ANOVA (and non-parametric) test and post-test used was Newman–Keuls multiple comparison test to find out the difference between OD value obtained in the sera of HC and leprosy patients (***p value < 0.0001, ** *p* value < 0.001, * *p* value < 0.05).

Highest mean OD value was obtained in the sera of T1R (0.416 ± 0.18) that was followed by LL (0.339 ± 0.13), ENL (0.322 ± 0.12), BL (0.302 ± 0.10), BB (0.275 ± 0.08), and TT/BT (0.264 ± 0.08). The mean OD value in sera of T1R patients’ group was significantly higher than TT/BT (*p* < 0.0001), BB (*p* < 0.0001), BL (*p* < 0.001), LL (*p* < 0.05), and ENL (*p* < 0.05) group of patients (Figure 1). The cutoff OD value for myosin was found to be 0.282. Seropositivity of antibodies against myosin in the sera of all types of leprosy patients is shown in Table 1. Highest percent of seropositivity was observed in T1R (75%) followed by LL (56.25%), BL (50%), ENL (46.66%), BB (43.47%), and TT/BT (35%). The seropositivity of patients with T1R was found to be significantly higher than TT/BT (*p* = 0.02) using Fisher’s exact test.

**Table 1:** Sero-positivity of anti-myosin antibodies in sera of leprosy patients and healthy controls (HC).

Subjects	HC	Leprosy patients					
	
	HC	TT/borderline tuberculoid	Borderline borderline	Borderline lepromatous	Lepromatous leprosy	Type 1 reaction	Erythema nodosum leprosum
Total number of individuals	45	20	23	26	32	20	15
Number of positive	3	7	10	13	18	15	7
Number of negative	42	13	13	13	14	5	8
Percentage positivity (%)	6.66	35*	43.47	50	56.25	75*	46.66

^*^p value < 0.05

### Lymphoproliferative Response of Leprosy Patients in the Presence of Host Myosin

The highest mean value of S.I. was obtained in T1R (4.06 ± 2.7) group of leprosy patients which was followed by BL/LL (2.71 ± 1.6), TT/BT (2.46 ± 1.3), and ENL (2.02 ± 1.4) patients.

**Figure 2:**
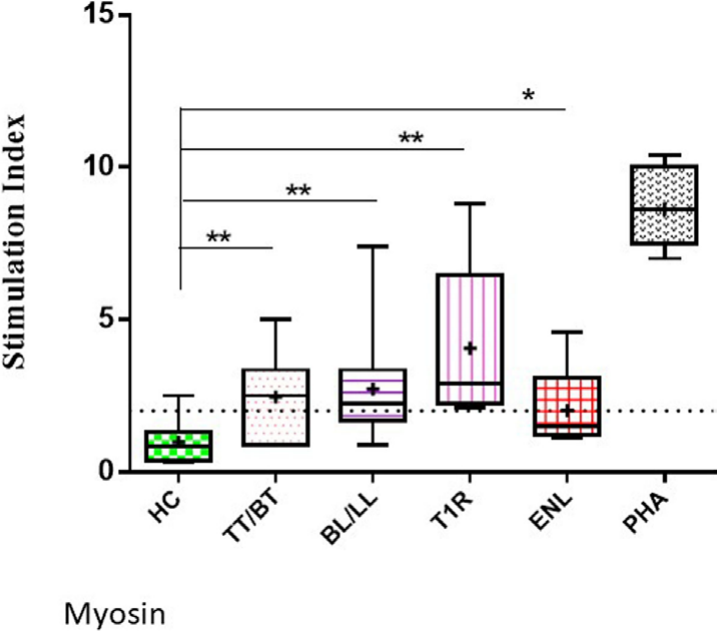
Level of lymphoproliferation in the presence of myosin in leprosy patient across the spectrum. Graphical representation is done by Box and Whiskers. Each bar represents the minimum to maximum values with median as the horizontal line and SD as error bars. +sign in each bar represent the mean value. Dotted line represents the S.I. = 2 (**p < 0.001, *p < 0.05).

The mean values of S.I. in the presence of myosin were found to be significantly higher in TT/BT (*p* = 0.005), BL/LL (*p* = 0.004), T1R (*p* = 0.004), ENL (*p* = 0.05) groups of leprosy patients in comparison to HC by using unpaired two-tailed *t* test (Figure 2).

### Levels of IgG Antibodies Against Myosin in MLSA-Hyperimmunized Rabbit

**Figure 3:**
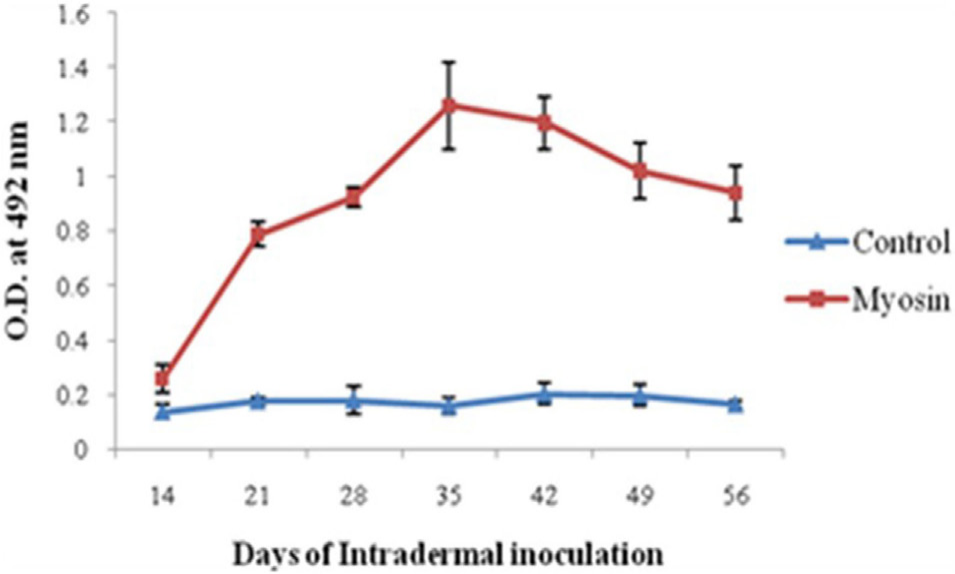
Levels of antibodies against myosin in *Mycobacterium leprae* soluble antigen-hyperimmunized rabbit sera at different time intervals. Each dot with error bar represents the mean OD value with SD at different time intervals.

Significantly higher levels of anti-myosin antibodies were observed in MLSA-hyperimmunized rabbit in comparison to control rabbit (average OD ± SD of MLSA hyperimmunized vs control 1.258 ± 0.16 vs 0.158 ± 0.03, *p* < 0.05). Highest levels of antibody against myosin (Figure 3) were observed at 35th day of immunization with MLSA.

### IgG Antibody Levels Against Myosin in MLSA-Hyperimmunized Mice

**Figure 4:**
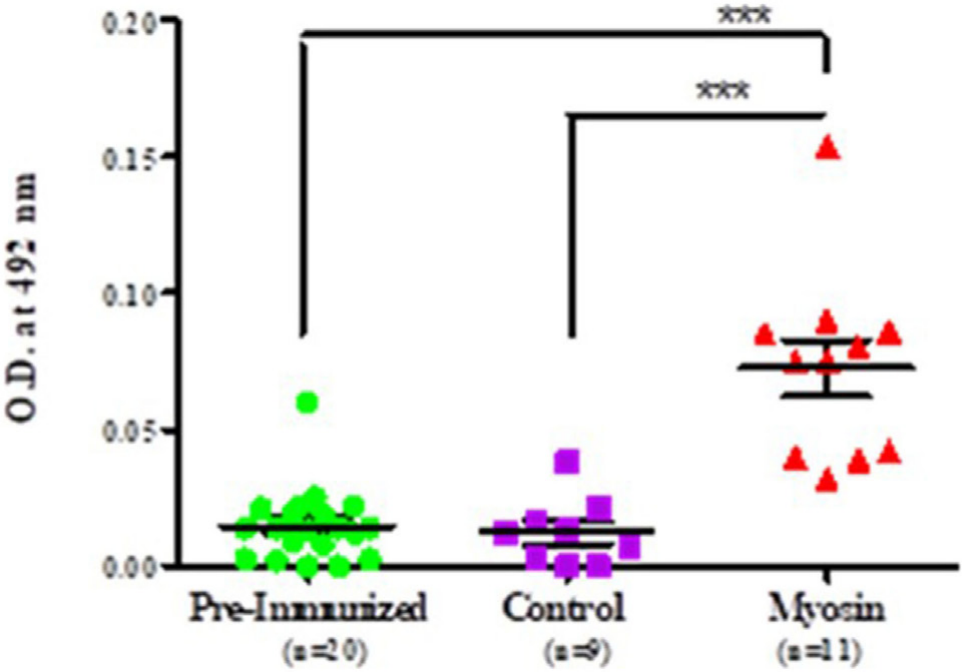
Comparison in levels of autoantibodies against myosin in sera of *Mycobacterium leprae* soluble antigen-hyperimmunized female BALB/c mice, pre-immunized mice, and control mice. Each dot represents individual OD obtained from mouse plasma. Solid horizontal line with error bars represent mean OD with SEM (***p < 0.0001).

It was observed that MLSA-hyperimmunized mice induce significantly elevated levels of anti-myosin antibodies in comparison to control mice (*p* < 0.0001). Mean level of antibodies against myosin was found to be significantly higher than pre-immunized (pre-immunized vs MLSA hyperimmunized 0.011 ± 0.009 vs 0.073 ± 0.035, *p* < 0.0001) and control group (control vs MLSA hyperimmunized 0.012 ± 0.012 vs 0.073 ± 0.035, *p* < 0.0001) at sixth week of inoculation with MLSA in female BALB/c mice by using one-way ANOVA (and non-parametric) and Bonferroni’s multiple comparison posttest (Figure 4).

### Adoptive Transfer With Immune Cells in Inbred Strains of Naïve Female BALB/c Mice

**Figure 5:**
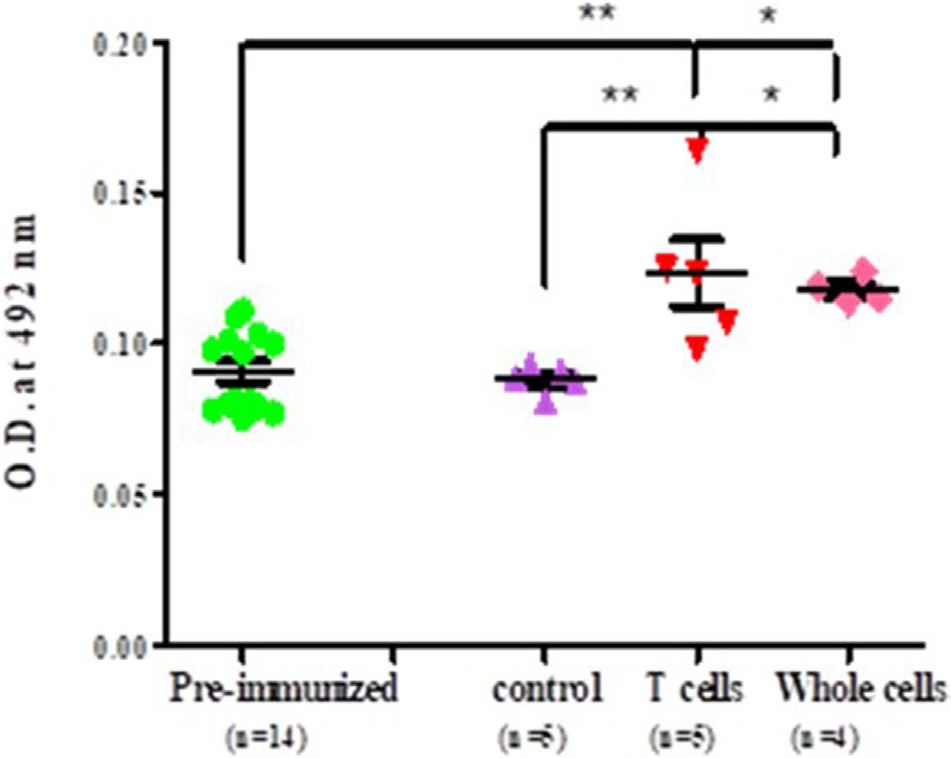
Level of antibodies against myosin proteins in adoptively transferred naïve female BALB/c mice. Each dot represents the individual OD. Horizontal line with error bars represent mean OD value with SEM. Control group adoptively transferred with whole cells and experimental group adoptively transferred with T cells and whole cells.

It was observed that significantly higher level of anti-myosin antibodies was observed in sera of adoptively transferred mice with nylon wool separated T cells (T cell vs pre-immunized 0.113 ± 0.0017 vs 0.090 ± 0.013, *p* < 0.001), splenocytes and lymph nodes cells (whole cell vs pre-immunized 0.1152 ± 0.027472 vs 0.0752 ± 0.004382, *p* < 0.05) in comparison to those of control and pre-immunized mice sera (Figure 5).

### Cross-Reactive Proteins Between Host Myosin and Mycobacterial Components

**Figure 6:**
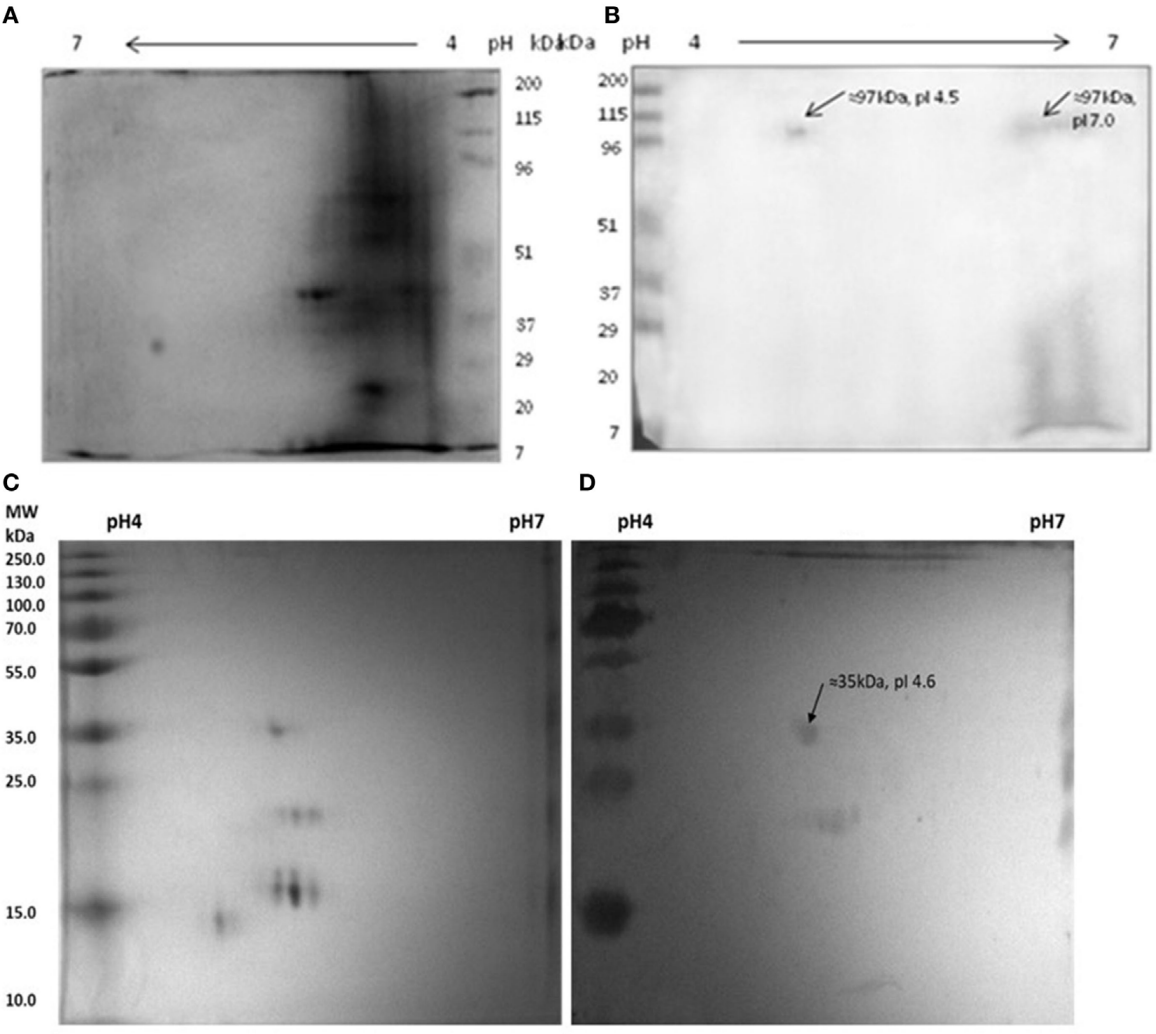
Reactivity of anti-myosin rabbit sera with *Mycobacterium leprae* soluble antigen (MLSA) **(A,B)** pooled leprosy patients’ sera with myosin **(C,D)**. **(A)** Protein profile of MLSA on 2-D gel stained with coomassie-blue, **(B)** Western blotting pattern of reactivity of anti-myosin rabbit sera with MLSA. **(C)** Protein profile of myosin on 2-D gel stained with coomassie-blue, **(D)** western blotting pattern of reactivity of pooled leprosy patients’ sera with Myosin.

It was observed that anti-myosin rabbit sera reacted with two isoforms of MLSA at ≈97 kDa, pI 4.5 and pI 7.0 (Figures 6A,B). Interestingly, we noted that pooled leprosy patients’ sera reacted with myosin at ≈35 kDa, pI 4.6 (Figures 6C,D).

### Identification of Cross-Reactive Proteins

The serum antibodies of leprosy patients reacted with tropomyosin alpha striated muscle isoforms (TM) (*Homo sapiens*) by MALDI analysis, whereas myosin-hyperimmunized rabbit sera that reacted with *Mycobacterium leprae* soluble antigen (MLSA) was identified as ATP-dependent C1p protease ATP-binding subunit (CLPC) of *M. leprae* (Table 2).

**Table 2:** Cross-reactive proteins identified by MALDI-TOF.

Cross-reactive Protein	Protein identified	Accession number	Mascot Score	Nominal mass	pI	Sequence coverage (%)
Myosin cross-reacted with pooledleprosy patients’ sera	Tropomyosin alpha striated muscle isoform (*Homo sapiens*)	AAT68295.1	93	32,690	4.67	42
MLSA cross-reacted with anti-myosin rabbit sera	ATP-dependent Clp protease ATP-binding subunit of *Mycobacterium leprae*	P24428	3	93,944	5.57	1

### Identification of Mimicking B-Cell Epitopes Between Tropomyosin and Probable ATP-Dependent Clp Protease ATP-Binding Subunit of *M. leprae*

**Figure 7:**
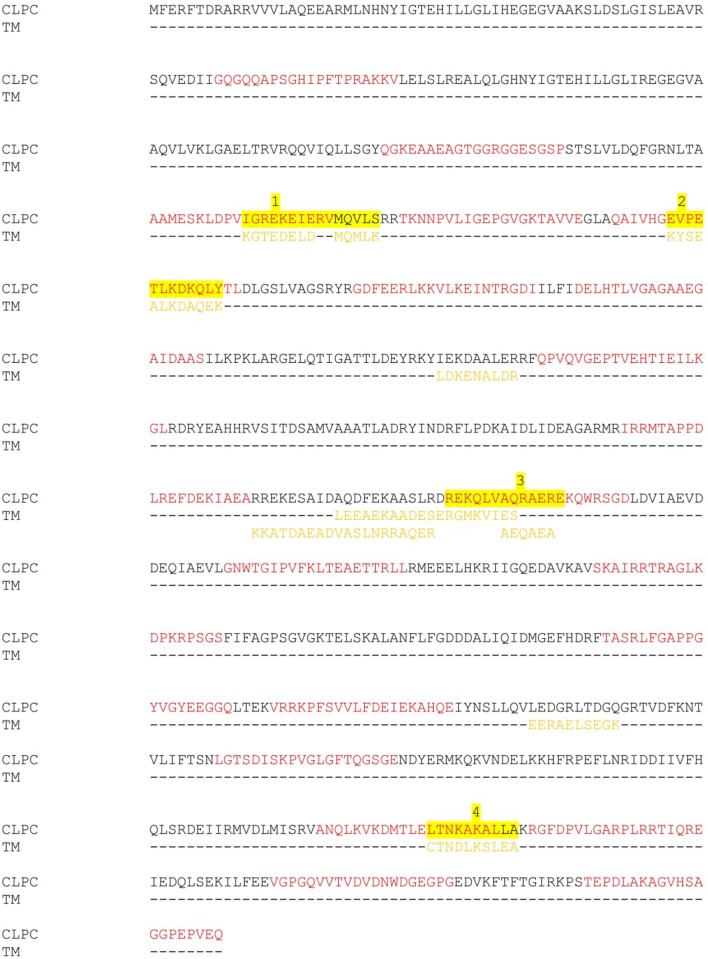
Multiple sequence alignment of Probable ATP-dependent clp protease ATP-binding subunit (CLPC) of *Mycobacterium leprae* and B cell epitopes of tropomyosin (TM) of host. Red color—showing predicted B cell epitopes of CLPC of *M. leprae*. Purple color—showing predicted B cell epitopes of tropomyosin of host. Yellow color—highlighted sequences showing mimicking B cell epitopes of both the proteins.

It was observed that four B-cell epitopes are mimicking epitopes of tropomyosin of host and probable ATP-dependent clp protease ATP-binding subunit of *M. leprae*. It was noted that CLPC_191–205_ with TM_41–48_ and TM_8–12_, CLPC_237–248_ with TM_49–60_, CLPC_453–465_ with TM_106–113_ and TM_23–28_ and CLPC_751–760_ and TM_161–170_ are putative mimicking epitopes (Figure 7).

### Three-Dimensional Structure of Tropomyosin and Probable ATP-Dependent Clp Protease ATP-Binding Subunit of *M. leprae*

**Figure 8:**
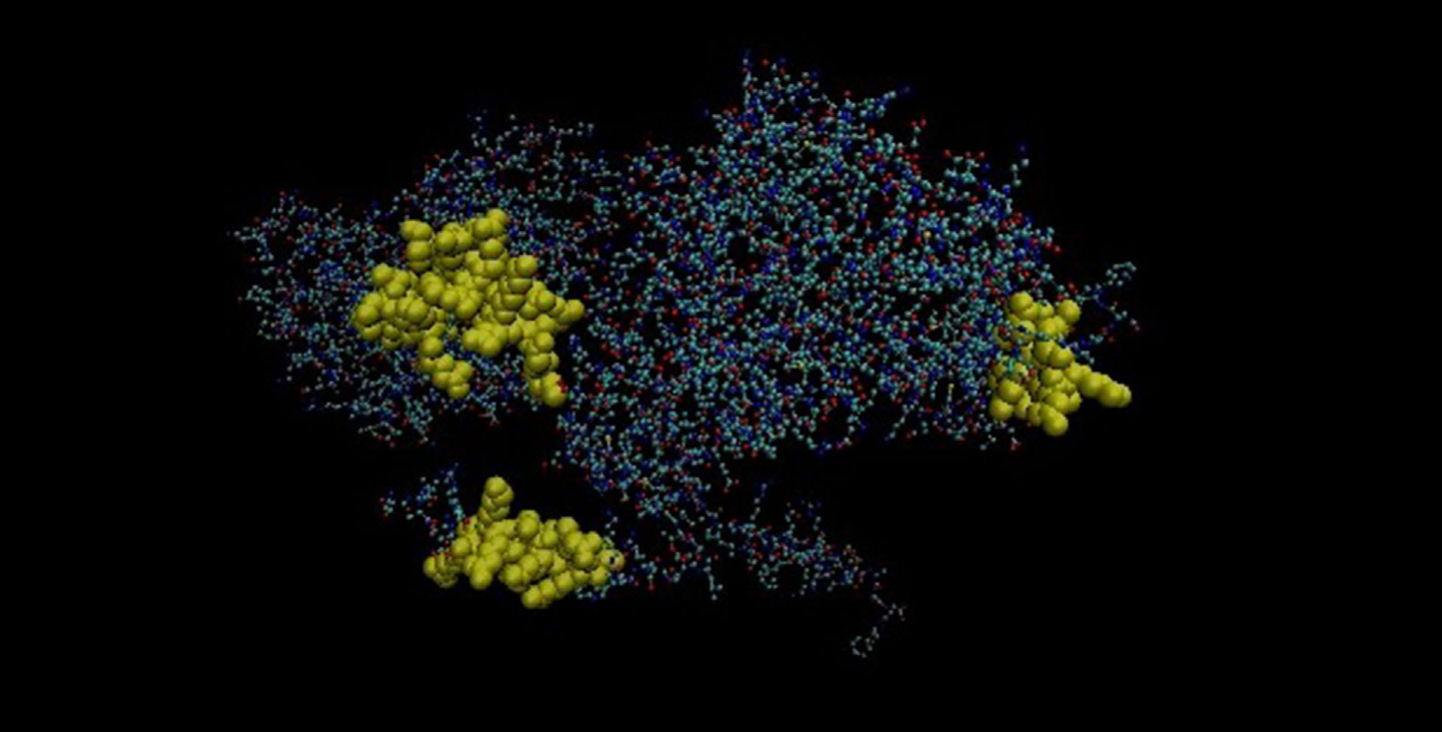
Three-dimensional structure of Probable ATP-dependent clp protease ATP-binding subunit of *Mycobacterium leprae*.

**Figure 9:**
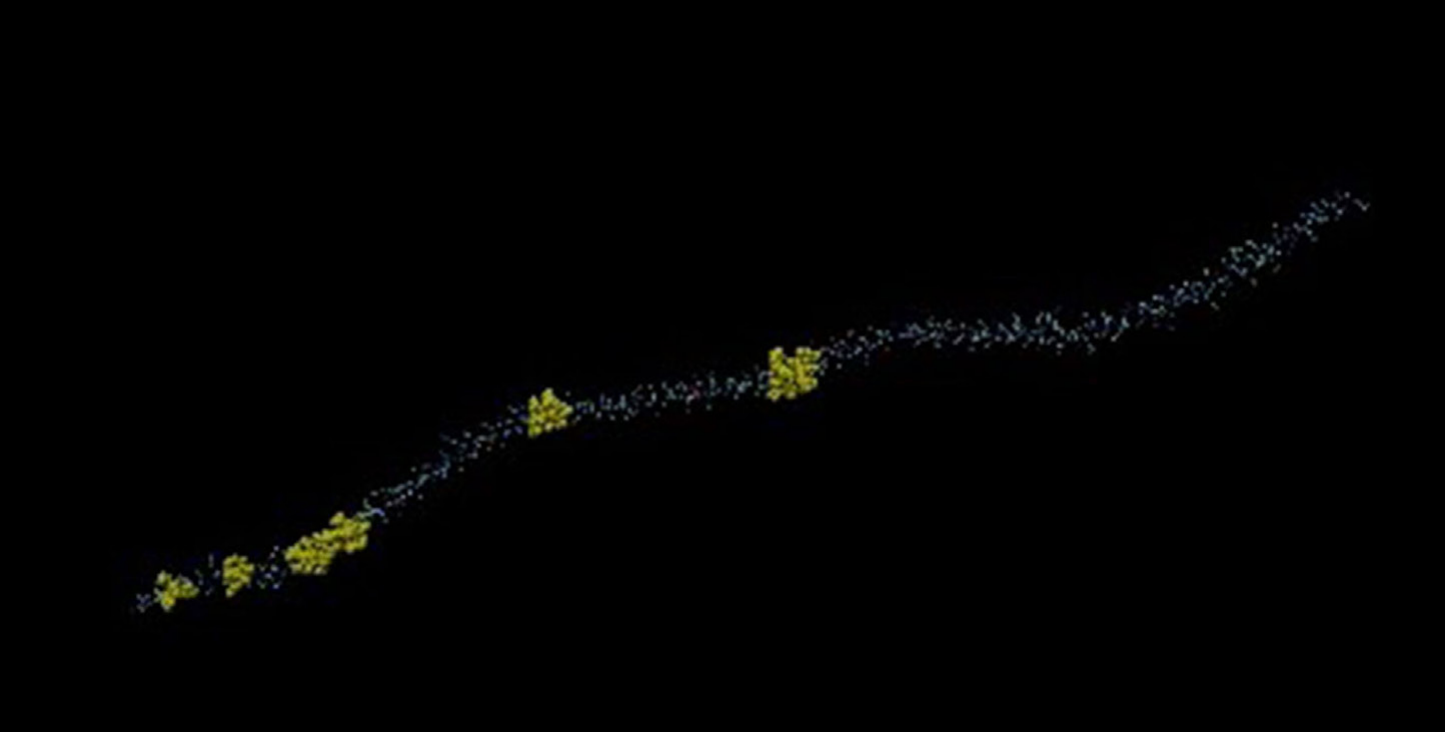
Three-dimensional structure of tropomyosin. Yellow area showing the mimicking epitopes.

Mimicking B-cell epitopes of both the proteins are highlighted on 3-dimensional structure of the proteins. It has been found that four putative mimicking B cell epitopes of CLPC of *M. leprae* and tropomyosin are present on the surface of the proteins (Figures 8 and 9).

## Discussion

In this study, we demonstrated the cross-reactivity of tropomyosin with sera of leprosy patients/*M. leprae* components using porcine myosin. The cross-reactivity is found in experimental animals also which are hyperimmunized with MLSA/porcine myosin. These results taken together suggest that common epitopes are shared between *M. leprae* and tropomyosin. Molecular mimicry is defined as epitopes shared between microbial antigens and host self-components (29) which may lead to autoimmunity, tissue injury and disease. We showed that significantly high levels of anti-myosin antibodies are present in all the groups of leprosy patients in comparison to HC. Highest level of anti-myosin antibodies was found in T1R (0.416 ± 0.18) which was followed by LL (0.339 ± 0.13), ENL (0.322 ± 0.12), BL (0.302 ± 0.10), BB (0.275 ± 0.08), and TT/BT (0.264 ± 0.08) (Figure 1). We observed significantly high lymphoproliferation with myosin in leprosy patients across the spectrum except ENL in comparison to HC (Figure 2). In the present study, porcine myosin was used to see the level of anti-myosin antibodies and lymphoproliferation in leprosy patients and experimental animals since, all the cytoskeletal proteins are conserved across the vertebrates and we observed high level of antibodies against this myosin in leprosy patients. We propose that molecular mimicry between putative epitopes of tropomyosin and *M. leprae* may potentially lead to loss of muscle functions in leprosy patients.

Leprosy is a chronic disease which affects both nerves and muscles. Leprosy is non-toxic disease and it was shown that most of the tissue and nerve damage occurs by host immune response to *M. leprae* antigens (30). Rambukkana et al. showed elegantly the immunological cross-reactivity between mycobacterial hsp 65 and human epidermal cytokeratin ½ (31). We recently reported existence of molecular mimicry between host cytokeratin-10 and HSP 65 (groEL2) of *M. leprae* (10) and between host myelin A1 and *M. leprae* 50S ribosomal L2 and lysyl tRNA synthetase proteins (11).

A central finding of this study is that MLSA induces antibodies against myosin in female BALB/c mice and this autoimmune reaction could be adoptively transferred to naïve mice. Hence, it supported our hypothesis that alteration in homeostatic mechanism may lead to autoimmune reaction and this autoimmunity is transferrable by autoreactive immune cells in naïve mice. Myosin reactive antibodies produced by immunization with MLSA could be adoptively transferred to naïve mice even by T cell transfer could be explained by the proliferation of autoreactive B cells present in the secondary lymph nodes which are known to induce autoimmunity (32). It is also noted that MLSA induces antibodies against myosin in rabbit. It was earlier observed that mouse cytomegalovirus infection induces myocarditis in susceptible BALB/c mice by producing autoantibodies to cardiac myosin and it was concluded that there were common epitopes between both the proteins (33).

Presence of autoantibodies are common in leprosy patients (34). A key question is whether these autoantibodies are produced because of mimicking epitopes between host protein/s and *M. leprae* protein/s. Significantly elevated level of antibodies against myosin is observed in leprosy patients across the spectrum in comparison to HC indicates that anti-myosin antibodies are produced because of the presence of some cross-reactive regions between both the proteins. Significantly high lymphoproliferation with myosin antigen is also noted in leprosy patients in comparison to HC. It is possible that high CMI level with host antigen might also be because of similarity of myosin protein with *M. leprae* protein/s. This study indicates that the cross-reactivity is at the 35 kDa of porcine myosin with leprosy patients’ sera and at 97 kDa of MLSA with anti-myosin rabbit sera. We propose that this cross-reactivity between myosin and MLSA may be because of presence of mimicking B cell epitopes in both the proteins. Further, these proteins are identified as tropomyosin of host and probable ATP-dependent clp protease ATP-binding subunit of *M. leprae*. We used porcine myosin for 2-D gel electrophoresis and western blotting but the reactive spot of porcine myosin with pooled leprosy patients’ sera identified by MALDI-TOF analysis was tropomyosin. We expected to find myosin as the reactive spot but it turned out to be tropomyosin, and this reactivity might be because of the presence of tropomyosin in the porcine myosin that reacted with pooled leprosy patients’ sera. Earlier reports from our group showing the presence of seven mimicking B cell epitopes of cytokeratin-10 and HSP 65 (10) and four mimicking B cell epitopes of myelin A1 and 50S ribosomal L2 and lysyl tRNA synthetase (11) were cross-reactive indicated their role in skin and nerve damage. Further, in the present study it is noted that four putative B cell epitopes are mimicking between tropomyosin and probable ATP-dependent clp protease ATP-binding subunit of *M. leprae*. These putative mimicking B cell epitopes might be responsible for “leprous myositis” leading to muscle damage in leprosy patients which has been reported earlier (17, 18). We have already reported in experimental mice that hyperimmunization with *M. leprae* antigen leads to lowering of Treg cells along with production of high levels of antibodies against *M. leprae* in addition to the production of high levels of autoantibodies against host proteins (10). Thus, these findings support our hypothesis that *M. leprae* infection can induce imbalance in homeostatic mechanism in immune system of the host and is responsible for the auto-reaction in leprosy patients.

For the first time, we identified the cross-reactive proteins between tropomyosin of host and probable ATP-dependent clp protease ATP-binding subunit of *M. leprae*. Further, it is noted that four B cell epitopes are putative mimicking B cell epitopes of both the proteins. We observed elevated level of antibodies against myosin and high level of CMI with myosin in leprosy patients in comparison to HC. The cross-reactive protein is at 97 kDa of *M. leprae* and at 35 kDa of myosin.

We also observed that this auto-reaction can be induced in experimental animals (rabbit and mice) after hyperimmunization with MLSA. This auto-reaction is transferrable to naïve mice with the help of immune cells. Hence, we conclude from our study that *M. leprae* infection can induce imbalance in the homeostatic mechanism of the host and can induce auto-reaction in leprosy patients. This induction in auto-reaction in leprosy patients is due to the presence of molecular mimicry between tropomyosin and probable ATP-dependent clp protease ATP-binding subunit of *M. leprae* which might be responsible for “leprous myositis” and muscular weakness.

## Ethics Statement

This study was carried out in accordance with the recommendations of “Indian Council of Medical Research guidelines, National JALMA Institute for Leprosy & OMD Human Ethics Committee” with written informed consent from all subjects. All subjects gave written informed consent in accordance with the Declaration of Helsinki. The protocol was approved by the “National JALMA Institute for Leprosy & OMD Human Ethics Committee”. This study was carried out in accordance with the recommendations of “guidelines of Committee for the Purpose of Control and Supervision of Experiments on Animals (CPCSEA),” “National JALMA Institute for Leprosy & OMD Animal Ethics Committee”. The protocol was approved by the “National JALMA Institute for Leprosy & OMD Animal Ethics Committee.”

## Authors Contribution

US conceived and designed the study; IS, KM, PS, VP, and AY performed the experiments and analyzed and interpreted the data; IS drafted the manuscript; KM, KK, DB, UG, and US critically reviewed the manuscript.

## Conflict of Interest Statement

The authors declare that the research was conducted in the absence of any commercial or financial relationships that could be construed as a potential conflict of interest.

## Footnotes

^1^http://www.matrixscience.com (Accessed: November 11, 2009 and Accessed: September 4, 2017).

^2^http://ailab.ist.psu.edu/bcpred/predict.html (Accessed: September 6, 2017).

^3^http://www.sbg.bio.ic.ac.uk/phyre2/html/.

^4^www.ks.uiuc.edu/Research/vmd/.
